# Single Cell RNA Sequencing of Rare Immune Cell Populations

**DOI:** 10.3389/fimmu.2018.01553

**Published:** 2018-07-04

**Authors:** Akira Nguyen, Weng Hua Khoo, Imogen Moran, Peter I. Croucher, Tri Giang Phan

**Affiliations:** ^1^Immunology Division, Garvan Institute of Medical Research, Darlinghurst, NSW, Australia; ^2^St Vincent’s Clinical School, Faculty of Medicine, University of New South Wales, Darlinghurst, NSW, Australia; ^3^Bone Biology Division, Garvan Institute of Medical Research, Darlinghurst, NSW, Australia; ^4^School of Biotechnology and Biomolecular Sciences, University of New South Wales, Kensington, NSW, Australia

**Keywords:** single cell RNA sequencing, memory B cells, dormant cancer cells, niche, two-photon microscopy

## Abstract

Single-cell RNA sequencing (scRNA-Seq) is transforming our ability to characterize cells, particularly rare cells that are often overlooked in bulk population analytical approaches. This has lead to the discovery of new cell types and cellular states that echo the underlying heterogeneity and plasticity in the immune system. Technologies for the capture, sequencing, and bioinformatic analysis of single cells are rapidly improving, and scRNA-Seq is now becoming much more accessible to non-specialized laboratories. Here, we describe our experiences in adopting scRNA-Seq to the study of rare immune cells in their microanatomical niches.

## Introduction

A major challenge in biology has been to identify and understand how the molecular changes that occur in a specific cellular population manifest as healthy, normal responses, or as disease. This has been particularly difficult in instances where the key cellular mediators are exceedingly rare, such as antigen-specific memory B cells in the lymph node, and dormant cancer cells in metastatic bone niches. Analysis of gene expression by bulk cell populations dilutes the contribution of these rare cells to the overall gene expression pattern. As a result, the complexity and diversity of the cells that are located in these niches and their unique molecular signatures are often lost. In addition, transcripts from relatively frequent contaminating cells can not only obscure the overall signature but can also be mistaken as the signature of the rare cells of interest. Single-cell analyses, on the other hand, have the potential to resolve these heterogeneous cell populations at an unprecedented scale (1, 2) and reveal unexpected hidden cell subpopulations (3, 4). This has been made possible by the advent of transformative single-cell technologies, such as fluorescence-activated cell sorting (FACS), microfluidic devices, and mass cytometry, which have enabled researchers to overcome the challenges posed by tissue heterogeneity (5). Recently, the development of photolabeling technologies such as two-photon photoactivation (6, 7) and photoconversion (8–10) have enabled researchers to precisely and optically mark rare cells in their microanatomical niches and isolate them for *ex vivo* analysis. Other technologies for linking single cell transcriptomes with spatial positioning include spatial transcriptomics, which is performed on tissue sections (11), Seurat which links the *in situ* hybridization patterns of a series of landmark genes to the single cell gene expression profiles to generate a probability map of the location of cells in the tissue (12), and transcriptome *in vivo* analysis, which uses photoactivation to capture RNA from cells in live tissue (13).

Advances in single-cell RNA sequencing (scRNA-Seq) have now made it possible to sequence the transcriptome of rare cells with small amounts of starting material. This has yielded large amounts of transcriptional information for the accurate, unbiased molecular characterization of these rare cells. Single cell transcriptomics provide crucial information that would otherwise be lost by bulk approaches; this is particularly important where well-established cell surface markers are neither known nor available for characterization by multiparameter FACS analysis or mass cytometry, or there is a large degree of heterogeneity within an apparently homogeneous cell population, such as rare antigen-specific B and T cells with clonal antigen receptors during the evolution of an immune response. This is a rapidly changing field in which new protocols and techniques are continuously being developed and improved. This review describes the experiences of a group of immunologists and bone biologists, with no prior knowledge or expertise in scRNA-Seq, in adopting the technology for our investigation of rare cells and the niches in which they occupy. Here, we outline the major considerations when embarking on an scRNA-Seq study: the design and experimental set up to acquire single cells, the preparation of single cells for sequencing, and analysis of the sequencing results. It is not a step-by-step protocol nor an exhaustive review of the tools and technologies currently available, but rather a practical guide to the technology that may help the beginner design, perform, and analyze scRNA-Seq experiments of rare immune cells [more detailed expert reviews are available, for example, in Ref. (14, 15)].

## Design of scRNA-Seq Experiments of Rare Cells

**Figure 1:**
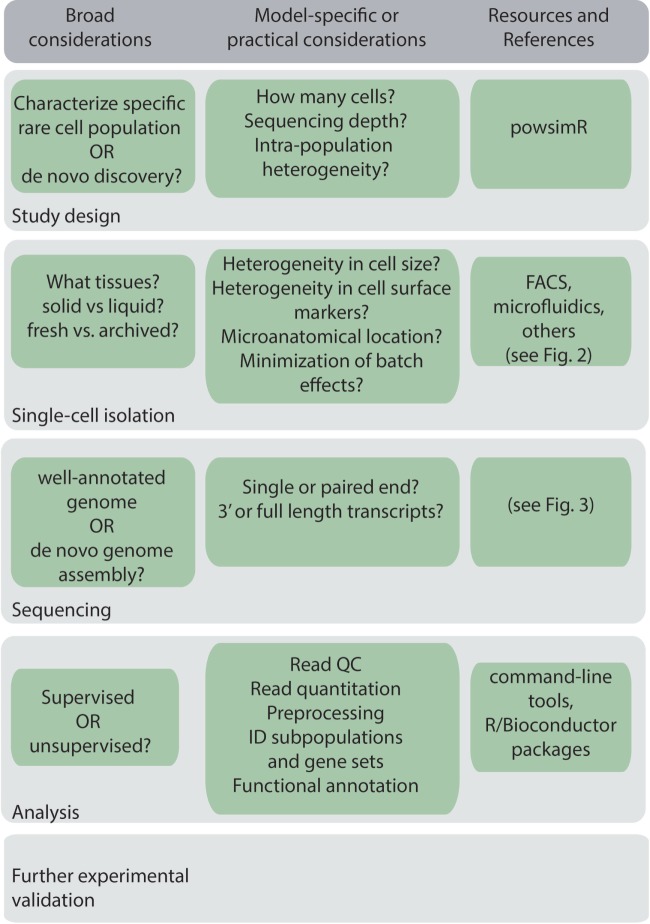
Key considerations in a general single-cell RNA sequencing workflow.

A general workflow for scRNA-Seq experiment is shown in Figure 1. Before beginning a scRNA-Seq experiment, it is important to plan out how many cells need to be sequenced, and the sequencing depth and coverage required to accurately detect and quantify lowly expressed genes (16). The amount of sequencing capacity used for a single sample, measured as the number of raw reads per cell, must be traded off against the sequencing cost. This will depend on the expected complexity, that is, the heterogeneity of the cells being sequenced and the degree of variability in their gene expression levels. Statistical packages, such as powsimR, are available to perform power calculations, which can be used to estimate the total number of cells that need to be sequenced (17). Sequencing depth also requires knowledge of the transcriptional activity of the cell and total mRNA content, which can vary significantly between, for example, resting and activated B cells, and dormant and proliferating myeloma cells. As a rough guide, half a million reads per cell was found to be sufficient for detection of most genes (18), although greater depth may be required for genes with low expression.

Another important consideration is the need to avoid technical bias through randomization of samples and minimizing batch effects if multiple experiments are performed at different time points, as it is difficult to completely computationally eliminate batch effects *post hoc*. Thus, preparing different experimental groups on library preparation plates and sequencing lanes and, if possible, minimizing batching of experiments is important in decreasing technical bias (19). Increasing the number of cells sequenced and the sequencing depth may also offset technical variability (20). Spike-ins, RNA molecules introduced into samples to calibrate measurements and account for technical variability are also essential, and typically utilize either External RNA Controls Consortium (ERCC) standards (21), or more recently Sequin standards, which align to artificial gene loci encoded within an accompanying *in silico* chromosome and better represent the complexity of eukaryotic gene expression and splicing (22).

## Identification and Preparation of Rare Single Cells

A key consideration when designing a scRNA-Seq experiment is whether to isolate a pure population of the cells of interest or a mixed population of cells containing the specific cells of interest. The strict *a priori* approach, where only the specific cells of interest are isolated, may be beneficial for well-characterized populations as this results in decreased heterogeneity of the sorted cells and thus may require less cells to be sorted and less sequencing depth. However, this strict approach may not reflect the underlying cellular or transcriptional diversity present in a population and may possibly introduce bias and exclude cells of potential interest. The latter, more agnostic, approach has additional benefits particularly in *de novo* discovery of new cell subtypes. For example, scRNA-Seq has identified new subpopulations of immune cells including innate lymphoid cell subsets (3) and dendritic cell and monocyte subsets (4) through sequencing a large number of cells that were enriched, but not specific to, these cell types. Thus, allowing some level of heterogeneity may be beneficial when designing a scRNA-Seq experiment, although this will impact on the number of cells that need to be sequenced, the depth of sequencing required, and the experimental cost. The more agnostic approach is also useful when isolating cells based on microanatomical location, as relaxing the sorting criteria has allowed identification of a more diverse range of cell types than may have been hypothesized (7, 23).

Methods to prepare single-cell populations of interest will be largely dictated in practice by one’s established experimental models and tissues of interest. Practical considerations include the type of tissue being analyzed, whether cells will be isolated immediately, or cryopreserved or fixed for later isolation. Many immunological organs of interest such as peripheral blood, spleen, or lymph nodes, are easily dissociated into a single cell suspension. However, complex solid tissues, such as tumors, often require mechanical or enzymatic dissociation. During preparation of single cell suspensions, cellular stress and death should be minimized to ensure that tissue preparation does not bias toward recovery of specific cell types. For example, some cell populations are more sensitive to heat stress than others. Furthermore, enzymatic digestion at 37°C and cell dissociation from solid tissues can introduce transcriptional changes in the cell, and this may be minimized by the use of cold active proteases from *Bacillus licheniformis* (24). Recent advances have found scRNA-Seq can also be performed on cryopreserved (25, 26) or fixed cells (27, 28) and cryopreserved cells were found to have a similar scRNA-Seq transcriptional profile to freshly isolated cells (25). These advances may also help minimize batch effects as they allow simultaneous processing of samples acquired at different times. Single nucleus RNA sequencing has also been developed to enable analysis of frozen or fixed tissues, and tissues that cannot be dissociated (29, 30).

Immunology research has typically relied on cell surface markers to identify cell populations of interest. This requires a well-characterized panel of markers that identifies the cell population of interest, while excluding unwanted cells that may complicate analysis. Cells can be excluded through use of singlet gates to remove doublets, dump gates with markers for unwanted cell subsets as well as dead cell exclusion markers. Another approach is to use fluorescent reporter mice, which enables identification of a specific cell population without the need for expression of defined cell surface markers. The fluorescent reporters can either be driven by specific promoters to mark a specific lineage or engineered to be co-expressed with any protein of interest. However, this approach precludes biological systems such as primary human tissue that cannot be genetically manipulated. If using mice, it is important to have a well-characterized mouse model.

In contrast to identifying cells based on expression markers, recent research has identified single cells based on microanatomical location. Fluorescent reporters that are either photoactivatable, such as photoactivatable-GFP (31) or photoconvertible, such as Kikume (32) and Kaede (33), allow precise optical marking of cells of interest by two-photon microscopy. By linking the photoactivatable or photoconvertable reporter to lineage-specific or antigen-specific fluorescent protein markers, cells can be precisely identified based not only on expression markers but also the localization to specific microanatomical locations in immune tissues. More recently, this approach was utilized to perform NICHE-seq and systematically characterize the cellular composition of the spleen, among other immune niches (7).

## Methods for Isolation and Collection of Rare Single Cells

The ability to isolate adequate numbers of viable single cells is one of the key determinants of a successful scRNA-Seq experiment. There are a number of methods available to isolate single cells for RNA sequencing.

### FACS Sorting

Isolation of single cells can be achieved through FACS sorting of single cells into 96- or 384-well plates. Often, intact single cells are deposited into a cell lysis buffer solution where the contents of the cell are released, and RNA preserved by RNase inhibitors within the solution. FACS sorting enables one to selectively sort cells of interest within a heterogeneous population based on expression of cell surface markers. However, FACS may not be ideal for extremely low volume samples such as fine needle aspirates, as there may be insufficient sample for cell staining, or for very rare cell populations, as isolation can be confounded by noise during FACS acquisition. If the population of interest is very rare, the cell sorting time may also be a limiting factor in the experimental design. In this case, it is often possible to pre-enrich by negative selection or depletion of the unwanted cell populations using magnetic-activated cell sorting (MACS), and then performing FACS sorting. This may increase the viability of the rare cell population as well as increase the feasibility of the experiment. An added benefit of FACS sorting is the ability to “index-sort,” that is, record surface marker protein expression levels for each cell and link this information to gene expression levels (34, 35). Index sorting enables one to retrospectively verify any correlations between scRNA-Seq data and expression of cell surface markers; this is particularly useful when cells have been stained and analyzed for a panel of markers but isolated agnostically, with minimal or no gating.

### Microfluidic-Based Approaches

Recent advances in microfluidics have enabled high-throughput droplet encapsulation-based methods to capture and barcode thousands of individual cells (36, 37). Cell suspensions are diluted to appropriate concentrations, calculated using Poisson distribution statistics, and single cells are captured in microwell or encased in water-in-oil droplets in a probabilistic manner. Within each microwell or aqueous droplet, cDNA generation is conducted at nanoliter reaction volumes, greatly reducing the volume of reagents required. This allows for low cost, high-throughput processing of hundreds to several thousands of cells. Common microwell encapsulation solutions commercially available are Fluidigm C1, Biorad/Illumina ddSeq, Clontech ICell8, and BD Rhapsody while droplet encapsulation methods include inDrop (36), DropSeq (37), and 10× Chromium from 10× Genomics. Microfluidics approaches are preferable when analyzing large numbers of cells. However, this may affect sequencing depth and hence power, so careful consideration must be given to the number of cells sequenced. Furthermore, the Fluidigm C1 system is currently restricted to relatively homogenous cell sizes and circular cell shapes, limiting its broader applications. Microfluidics approaches generally lack the ability to differentiate cells based on fluorescent reporters or cell surface markers, so to isolate a rare cell population, it may be necessary to enrich the population of interest, through MACS or FACS, prior to microfluidics approaches. This is optimal only if there are sufficient cells (typically at least thousands) recovered for input into the encapsulation systems, something that may not always be possible. Low capture efficiency remains a limitation of encapsulation-based systems, as this restricts the ability to isolate rare populations from samples with low cell numbers; most of these cells will not be captured, leading to selection bias in the data. Capture efficiency is influenced by the concentration of the cell suspension and the flow rate and is modeled by Poisson distribution probabilities (38). High cell concentrations maximize the throughput of encapsulation of cells in droplets (i.e., the probability that all droplets contain cells), but only 37% of these droplets will contain single cells, while 42% are estimated to contain more than one cell. Conversely, low cell concentrations reduce the encapsulation such that only 5% of all droplets contain cells, but 98% of these will contain only single cells. Thus, deterministic encapsulation strategies that identify and isolate droplets containing single cells have been in development to overcome the limitations of Poisson distributions (38). Different strategies and engineering approaches have been taken across various commercial encapsulation systems to optimize the capture efficiency of single cells. For example, the inDrop system has a reported 7% singlet capture efficiency (36). Other commercial microwell encapsulation systems such as Fluidigm C1, Biorad’s ddSeq, and Clontech’s ICell8 have an estimated singlet capture efficiency ranging from 2.6 to 39%, while 50% singlet capture was reported using droplet-based Chromium (26). Regardless of the approach adopted to isolate single cells, care must be taken to avoid the capture of doublets, whereby two or more cells are captured in a well or droplet, as this will confound the scRNA-Seq results. FACS exhibits relatively low doublet rates of 2.3% (39) while up to 30% has been reported using microwell encapsulation systems (37). It is claimed that the ability to image and visually inspect cells post-capture to control for empty wells or doublets on the Clontech ICell8 and recently improved Fluidigm C1 platforms have reduced the doublet rate to 3%.

### Other Methods

An alternative method of cell isolation is laser capture microdissection, for tissues that are less amenable to single-cell suspension (40). This is particularly useful for archived pathology samples, such as formalin-fixed paraffin-embedded tissues. While it has the benefit of not needing to dissociate cells, this approach generates a low yield, particularly for low abundance RNA species, and is low throughput. When cell numbers are very limited or when cells are too fragile to undergo other methods of isolation, micro-manipulation is another low throughput way of manually collecting individual cells while minimizing cellular stress. In this way, for example, viable circulating tumor cells can be isolated from the blood of a melanoma patient by combining micro-manipulation with fluorescence microscopy (41). Although micro-manipulation is labor-intensive and slow, throughput can be improved using robotic automation solutions such as the CelllenONE system (Cellenion).

## ScRNA-Seq Library Preparation

**Figure 2:**
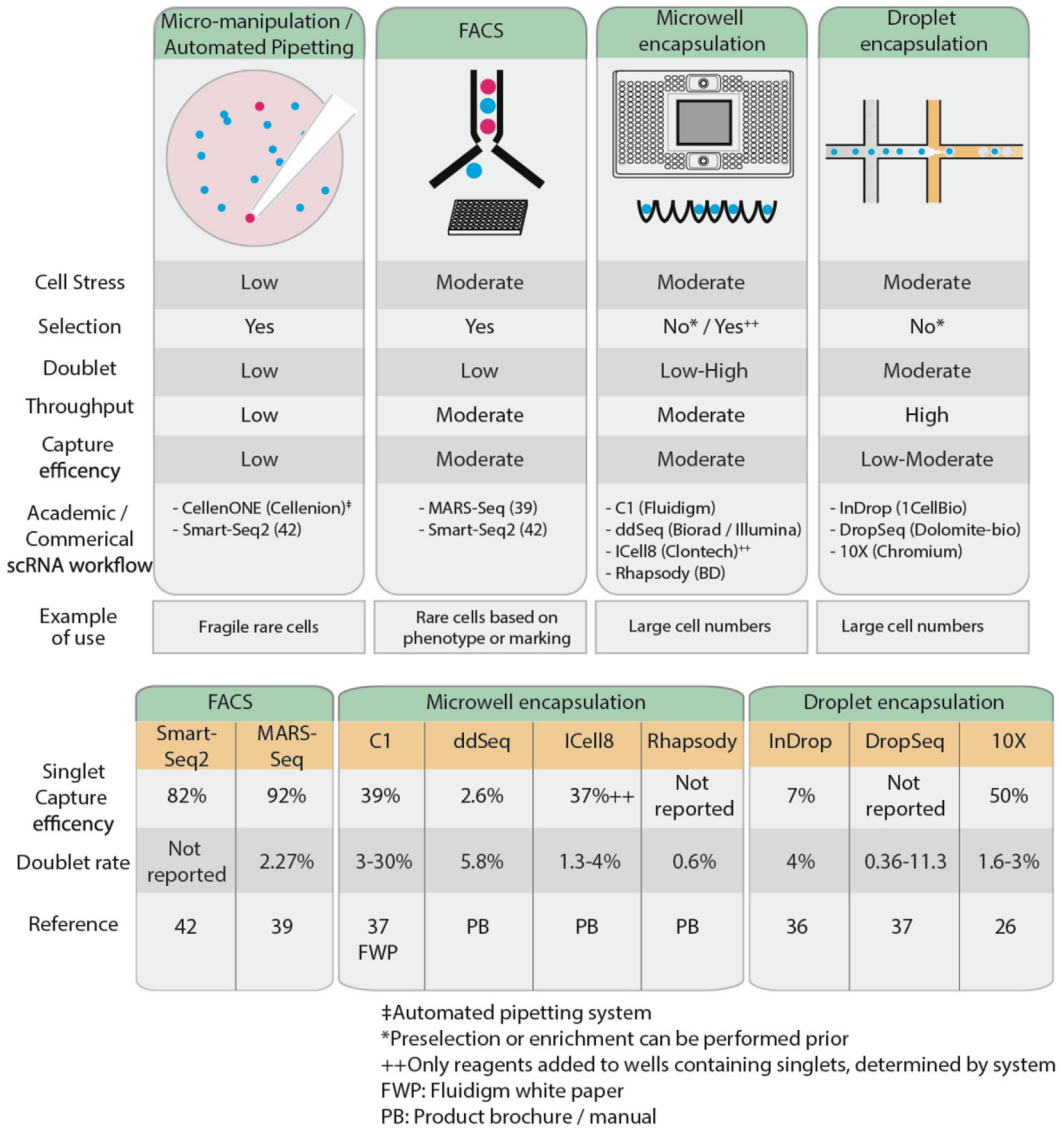
Comparison of the characteristics of several common single cell isolation methods (top), reported single cell capture efficiency and doublet rates (bottom).

Following single-cell isolation and collection, samples are processed to generate cDNA prior to single-cell sequencing library preparation. The exception to this is samples from microfluidics platforms, where cDNA generation is integrated at the sample collection stage. Another consideration to keep in mind when designing a scRNA-Seq workflow is the type of sequencing to carry out, which is intimately linked to the research question at hand. The cell isolation method adopted may limit the sequencing platform and the type of sequencing library that can be prepared, and hence also the scRNA-Seq application (Figure 2).

**Figure 3:**
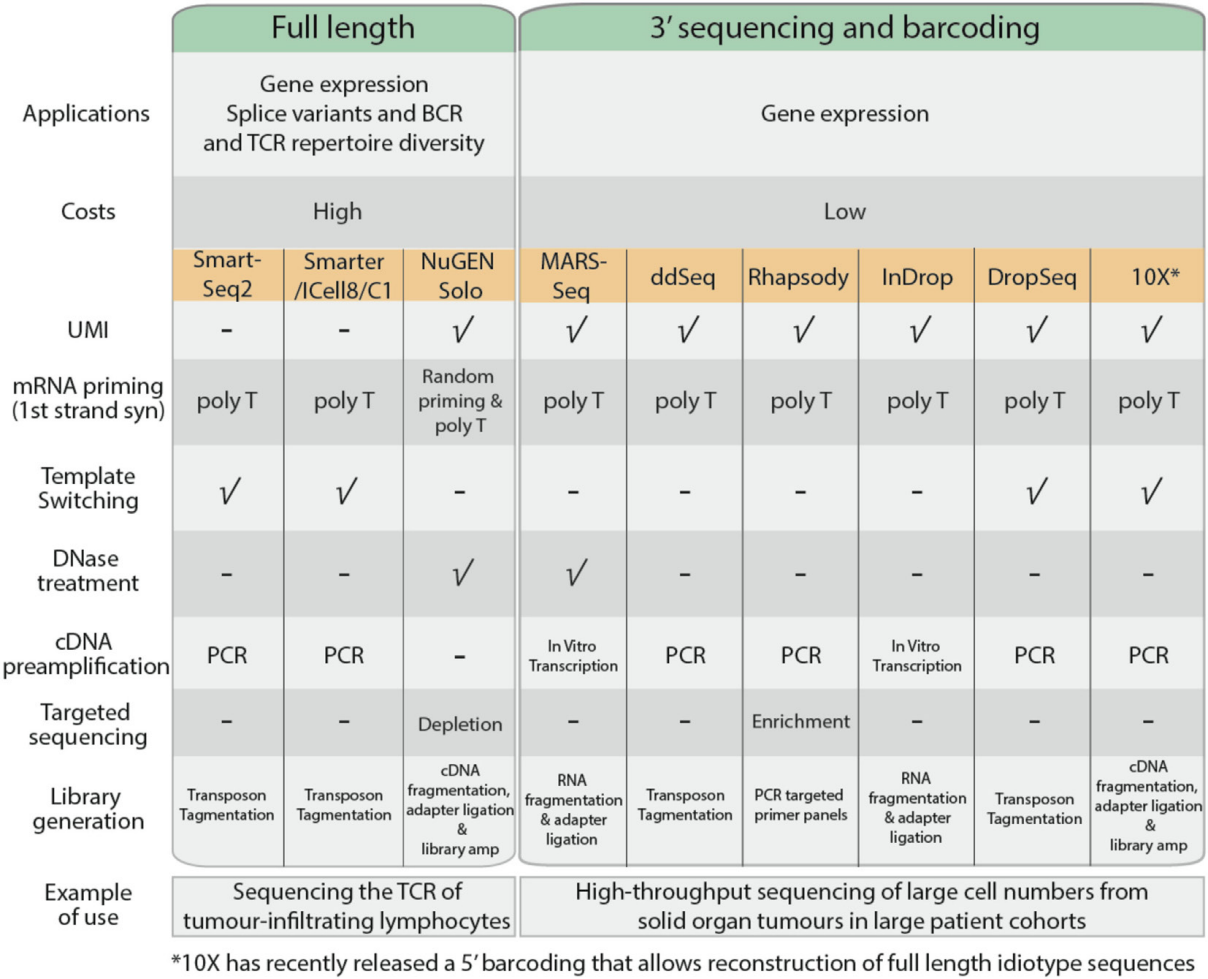
Comparison of common single cell RNA sequencing protocols, features, and their applications.

Single-cell RNA sequencing can be used to measure gene expression, detect spliced transcript variants, or to determine the unique immunoglobulin and T-cell receptor (TCR) profiles of individual cells. In general, most common scRNA-Seq protocols adopt either full-length or 3′ end sequencing (Figure 3). All the applications mentioned above can be performed using full-length sequencing where the complete cDNA is generated and sequenced. Full-length sequencing also enables greater detection of low abundance transcripts (42). However, due to the higher depth of sequencing coverage required, the cost of full-length sequencing is much higher. In contrast, for 3′ end sequencing, approximately 70–100 bases from the 3′ end of the transcript are sequenced instead of the complete transcript. This allows greater sample pooling of thousands of cells within sequencing runs, greatly reducing the costs per cell. However, despite the lower costs involved, 3′ end sequencing approaches are only useful for quantification of gene expression.

The cDNA generation stage begins with the capture of poly-A mRNA molecules using poly-T primers to avoid capture of genomic DNA and rRNA. In 3′ sequencing workflows, the poly-T primers consist of unique molecular identifiers, a series of 4–12 random nucleotide sequences that uniquely tag an individual mRNA copy of each transcript (43). This improves quantification of each transcript based on the number of UMIs, allowing for adjustment of biases that arise during product amplification (44). Full-length sequencing approaches such as SmartSeq2 (45) and SMARTer (Clontech) adopt template-switching strategies to generate full length cDNA. Upon reaching the 5′ end of the RNA template, MMLV reverse-transcriptase incorporates a few bases to the 3′ end of the newly synthesized cDNA strand and switches to the nascent strand to complete replication upon base-pairing with the complementary template-switching oligo. Using template-switching strategies, poly-T primer and template-switching oligos flanking nascent cDNA are amplified, leaving genomic DNA and truncated mRNA unprocessed. Some scRNA-Seq protocols such as MARS-Seq and NuGen Solo also incorporate a DNase treatment step to remove contaminating genomic DNA present within the sample.

The quantity of cDNA obtained from individual cells is dependent on the transcriptional activity of the cell and its mRNA content, which is approximated to be 10 pg per cell (41). The cDNA is amplified using PCR or *in vitro* transcription to generate sufficient material for sequencing. PCR is a commonly used approach to exponentially generate copies of cDNA; however, there is the potential to selectively overamplify high-abundance transcripts and introduce bias in the data. *In vitro* transcription overcomes this limitation as nascent cDNA containing T7 promoter sequence is linearly amplified by T7 RNA polymerases.

The transcriptome of a cell has a high dynamic range, where a minority of highly expressed genes such as ribosomal genes constitute the majority of RNA molecules (21). These highly expressed genes will occupy most of the sequencing reads, while the sequencing coverage of lowly expressing transcripts will be sparse, affecting accurate quantification. Protocols by BD Rhapsody and NuGen Solo promote targeted sequencing, either through enrichment of genes of interest or removal of highly expressing ribosomal genes before sequencing, thereby providing an enrichment of sequencing read coverage (46).

Fragmentation and adapter incorporation of the single cell libraries is required for sequencing on the Illumina short read sequencing platforms. Most protocols adopt transposons to fragment the sequencing libraries while others adopt mechanical (NuGEN Solo), enzymatic (10× Chromium), or chemical (MARS-Seq and InDrop) approaches. This is followed by a limited PCR amplification step to incorporate Illumina adapter sequences to generate sequence ready libraries.

## Analysis: Extracting Biological Significance from Raw scRNA-Seq Data

Single-cell RNA sequencing generates tens to hundreds of gigabytes of data, in fastq format. The single-cell sequencing libraries will have been indexed or barcoded and pooled for sequencing, so, the raw data will need to be demultiplexed so that there is one fastq file per single-cell sample for single-end sequencing, or two fastq files per single-cell sample for paired-end sequencing. Ultimately, a typical scRNA-Seq experiment generates hundreds to thousands of fastq files, and a high-performance computing cluster is recommended to handle such a volume of data. There are many command-line tools and R/Bioconductor packages available, with more in development, to analyze scRNA-Seq data. Here, we outline the steps and suggest some packages to provide a framework for analysis or a foundation on which to base discussions with bioinformaticians surrounding analysis.

### Sequence Read Quality Control

Before delving into the data to uncover the underlying biology, one must reduce technical noise by processing raw reads through several quality control steps. Sequence read quality, adapter and GC content, length, and over-represented sequences can all be assessed and summarized using *FastQC* (47). Any adapter sequences should be trimmed off, using *Cutadapt* (48) or *Trimmomatic* (49) so that they do not adversely affect mapping to the reference genome or transcriptome. Quality trimming is generally not recommended for RNA-Seq, unless the sequence quality score is very poor (below Q10). Short reads, less than 100 bp, should also be discarded (50) as they can be difficult to map or can map to more than one locus, leading to false positive mapping and inaccurate read quantification. Shorter reads can be accommodated for more targeted sequencing applications such as TCR gene sequencing; a recent systematic study of the impact of sequencing depth and read length on data quality found that short reads up to 50 bp could be used to successfully reconstruct the TCRαβ receptor, but efforts to do so using 30 bp reads were unsuccessful (51).

### Alignment and Quantitation

To quantify gene expression, the reads need to be mapped to a reference database, and this can be achieved using tools originally designed for bulk RNA-Seq applications. A key consideration is whether to map to the genome or transcriptome. For organisms such as mouse or human, where transcriptome annotation is comprehensive, mapping to the transcriptome may increase unique, unambiguous mappings and be faster. However, this approach precludes novel gene discovery. For such purposes and for organisms with new or uncharacterized transcriptomes, it may be better to map to the genome. Direct alignment and splice-aware tools such as *Tophat* (52), *STAR* (53), or *HISAT* (54) create SAM output files that can be converted to BAM for visualization on *Genome Browser* or *Integrative Genome Viewer* (IGV). Alternatively, one can try pseudoalignment tools such as *Kallisto* (55), *Salmon* (56), and *Sailfish* (57). These tools rely on mapping k-mers (e.g., 7-mers) derived from the sequence reads to the reference index, avoiding alignment of individual bases and are hence computationally less demanding.

As mentioned earlier, inclusion of spike-ins such as ERCC or Sequins just prior to cDNA preparation enables measurement of technical variation and facilitates correction of batch effects. To quantitate spike-in abundance in each single-cell sample, it is necessary to generate a co-index, by concatenating the reference genome or transcriptome and spike-in sequences. Alignment or pseudoalignment is carried out to this co-index to determine read abundance.

### Preprocessing

Once a matrix of raw count data has been obtained, single-cell specific analysis tools are required to identify differentially expressed genes. Single cell data is sparse, with many genes exhibiting zero expression values, either due to the dynamic nature of transcription and resultant temporal fluctuations in gene expression, or due to “dropout” events, where inefficient reverse transcription during library preparation results in undetectable gene expression for low abundance genes. Consequently, the number of observed expressed genes in single cell data is lower compared to the average of a bulk population and there is more intra-population heterogeneity (58). Statistical assumptions about distributions that apply to bulk RNA-Seq data do not apply to single-cell data. Several R/Bioconductor packages and statistical methods have been developed, modeling distributions more relevant to single cell data, which is important for accurate normalization and identification of differentially expressed genes.

The *scater* package is a single-cell analysis toolkit that enables one to perform preprocessing, QC, simple normalization, and data visualization (59). To prepare the dataset for downstream analysis, identify and discard cells with high proportion of spike-in content, as this is symptomatic of endogenous RNA degradation or high-proportion of dropout events, filter out low abundance genes, and normalize for library size. Within *scater*, one can reduce the dimensions and visualize the data using principal components analysis (PCA) (60) or *t*-distributed stochastic neighbor embedding (t-SNE) (61) and perform more exploratory analyses to identify any confounding factors such as batch effects or cell cycle effects and correct for them. A more comprehensive normalization protocol more suited to single-cell data can be accessed through *scran*, which integrates seamlessly with *scater*, and has been shown to outperform existing methods such as trimmed mean of *M*-values normalization (TMM) (62). *Scran* pools cells of similar library sizes sums expression values across these cells to effectively reduce 0s, and determines pooled “size factors,” which are then deconvolved to infer individual cell size factors for more accurate normalization.

### Differential Expression and Clustering Analysis

Preprocessing ensures that downstream analyses are valid, and that any differential expression is not an artifact of gross technical variations such as sequencing depth or batch effects. However, there are more subtle sources of variation such as transcriptional bursting (63) and amplification bias. The purpose of any differential expression analysis is to robustly uncover molecular elements and pathways that underpin different biological processes and cellular states. Where populations or cellular states are known *a priori*, or defined in the experimental design, differentially expressed genes can be identified using supervised statistical methods such as *Single Cell Differential Expression* (*SCDE*) (64), or *Bayesian Analysis of Single-Cell Sequencing Data (BASiCS)* (65). *SCDE* operates on a mixture of Poisson distributions to estimate error, dropouts, amplification biases, and negative binomial distributions to model detected transcripts. Similarly, *BASiCS* is an integrated Bayesian method based on a two Poisson-gamma model to decompose variation into technical and biological components.

One of the key strengths of scRNA-Seq is the capacity for *de novo* discovery of new cell types or states. This is essentially a problem of unsupervised cell clustering, where rank *k* number of populations exists in the data. The rank *k* is resolved by machine learning and dimension reduction methods applied to the expression matrix of the most variable genes. Rather than using any one analysis, we strongly recommend trying a variety of analyses and seeing if results concur.

Highly variable genes to be fed into unsupervised clustering pipelines can be identified using *M3Drop* (66), which is based on the idea that dropout events are a consequence of inefficient reverse transcription, i.e., an enzymatic process, and hence can be modeled by Michaelis–Menten enzyme kinetics. Furthermore, there is a strong non-linear relationship between the dropout rate and the mean expression. Significantly variable genes are identified as being outliers to the right of the Michaelis–Menten curve. A matrix of *m* highly variable genes across *n* single cell samples can then be analyzed further to identify subpopulations and associated gene signatures or “metagenes” using dimension reduction methods such as PCA or non-negative matrix factorization (NMF) (67).

#### Principal Components Analysis

In PCA, all the data are projected in hyperdimensional space and a linear transformation is applied such that the greatest variance is captured in the first few new axes or “principle components” (PC). The contribution of each gene to each PC can be extracted from the loadings, although this is not always obvious because PCA operates in negative and positive space. Furthermore, PCA assumes linear and normally distributed data, which may not necessarily be applicable to the matrix of highly variable genes from the scRNA-Seq data (68).

#### Non-Negative Matrix Factorization

Non-negative matrix factorization is another linear decomposition method, but unlike PCA, it has non-negative constraints, allowing only additive combinations, which lends itself to parts-based representations (67, 69). As it interprets a dataset as a superposition of distinct parts, one can intuitively determine which genes define any particular subpopulation. When applied to scRNA-Seq data, it has been shown to outperform PCA (70). NMF was recently applied to a scRNA-Seq dataset of 5,902 cells from 18 head and neck squamous cell carcinoma patients, including five matched pairs of primary tumor and lymph node metastases, to characterize intratumoural heterogeneity and identify gene expression programs associated with metastatic disease (71). This identified an NMF metagene that lacked classical epithelial-to-mesenchymal transition (EMT) transcription factors, deeming it a “partial-EMT” program, which was expressed in a subpopulation of malignant cells. They performed immunohistochemistry using these newly identified markers and found that cells expressing a partial-EMT program were localized to the leading edge of invasive tumors. Additionally, they found that expression of this partial-EMT signature was predictive for nodal metastasis.

#### t-SNE

t-SNE is a non-linear method, which is gaining popularity and requires the user to set a rather arbitrary “perplexity” parameter, which defines the number of neighbors to use to build a nearest-neighbor network and determines the balance between preservation of local versus global structure. Samples are then clustered by random walks on the nearest-neighbor network such that local distances between cells are minimized. The stochastic nature of this method means that results will vary from run to run. Furthermore, distances are not linear and therefore relatedness cannot be inferred directly. It is recommended that the t-SNE algorithm be run multiple times at different perplexities to check the stability of the results, and that it be used for data visualization purposes rather than dimension reduction (61).

#### SC3

Specifically designed for scRNA-Seq data, *Single-Cell Consensus Clustering* (SC3) (72) is a user-friendly and interactive package for unsupervised clustering. It relies on the *k*-means clustering algorithm, iteratively assigning cells to *k* number of cluster centers or “centroids” based on minimal distances of mean values. Centroids are then recomputed to maximize local density of single-cell samples. With the capacity to test a range of *k* values in parallel, it is computationally intensive but has been shown to outperform t-SNE and PCA (72). For example, to define the molecular basis of susceptibility to fatal avian H7N9 influenza virus, investigators analyzed the TCRαβ repertoire and transcriptomes of circulating CD38^+^, HLA-DA^+^, PD-1^+^, CD8^+^ T cells from infected patients (73). Samples were collected at early and late timepoints from patients who had succumbed to the disease and compared to that of patients who had survived. By PCA, the samples segregated across the two patient groups, particularly at the early timepoint. Detailed analysis using SC3 enabled the authors to identify 279 differentially expressed genes, including PDCD5, PSMA5, and the Heat Shock Protein DNAJB1, across four clusters. The SC3 analysis highlighted the increased transcriptional heterogeneity among T cell clonotypes over time from fatal patients and suggests that CD8+ T cells in fatal patients are programmed to engage rapidly in antiviral responses rather than antigen-specific TCRαβ-mediated responses.

#### Monocle

Another single-cell focused application is *Monocle*, which enables clustering by pseudotime (74), that is, according to progression through a cellular process such as differentiation or oncogenic transformation. This can be done even in the absence of time series data. Consider a population of cells captured at the same time. Each cell represents a distinct stage and time-point along a continuum. *Monocle* learns the sequence of gene expression changes each cell must go through as part of a dynamic biological process and projects the cells along that trajectory. It can then identify genes that are regulated over the course of the trajectory. For example, the mechanism of chromosome 7 loss in myeloid malignancies was recently mapped using *Monocle* to show that hematopoietic stem and progenitor cells from patients with monosomy 7 had distinctly different differentiation trajectories, as well as gene signatures that were indicative of dysregulated immune response, DNA damage checkpoint, and apoptosis pathways compared to healthy controls (75).

Once gene signatures have been identified for the cell populations of interest, functional annotation analyses to gain insight to the biological significance of the gene sets can be performed using any of the well-established gene ontology tools utilized in microarray or RNA-Seq gene expression studies.

## Conclusion

A well-designed scRNA-Seq experiment can empower studies of rare cell populations, particularly in efforts to understand physiological or disease processes. Here, we have outlined the key considerations in designing a scRNA-Seq workflow, from sample acquisition through to data analysis. Given the rarity of some immune populations and the limitations on sample size this may impose, careful deliberation and extra attention should be given at every step of the way to ensure that the study is sufficiently powered to detect even subtle molecular changes that may take place. Furthermore, the process of scientific discovery clearly does not stop at a list of candidate genes, but the scarcity of cellular material from rare single-cell samples makes experimental validation somewhat difficult.

## Author Contributions

AN, WK, and IM are equal first authors. AN and TP are co-corresponding authors. AN, WK, IM, PC, and TP all contributed to the writing of this manuscript.

## Conflict of Interest Statement

The authors declare that the research was conducted in the absence of any commercial or financial relationships that could be construed as a potential conflict of interest. The handling Editor declared a shared affiliation, though no other collaboration, with the authors.
